# Development and temporal validation of 1-, 2-, and 3-year survival prediction models for hepatocellular carcinoma using the SEER database

**DOI:** 10.1038/s41598-026-48480-9

**Published:** 2026-04-11

**Authors:** Zixuan Fu, Keru Hou, Yaowu Zhao, Zhouyan Wang, Yuhao Qiu, Qingyuan Yao, Ping Huang

**Affiliations:** 1 Department of Hepatobiliary Surgery, First Affiliated Hospital of Chongqing Medical University, Chongqing, People’s Republic of China; 2 Department of Respiratory Medicine, National Clinical Research Center for Children and Adolescents’ Health and Diseases, Ministry of Education Key Laboratory of Child Development and Disorders, Children’s Hospital of Chongqing Medical University, Chongqing, People’s Republic of China

**Keywords:** Hepatocellular carcinoma, SEER program, Survival analysis, Cox proportional hazards models, Nomogram, Risk stratification, Biomarkers, Cancer, Computational biology and bioinformatics, Gastroenterology, Oncology, Risk factors

## Abstract

Hepatocellular carcinoma (HCC) accounts for most primary liver cancers and displays substantial variability in survival outcomes. Existing staging systems, such as the American Joint Committee on Cancer (AJCC), Barcelona Clinic Liver Cancer (BCLC), and China Liver Cancer Staging (CNLC) systems, often fail to capture individual heterogeneity. Additionally, many published prognostic models rely on small cohorts, predict survival at only a single timepoint, or lack temporal or external validation. Using data from the Surveillance, Epidemiology, and End Results (SEER) Program, we developed and temporally validated multi-timepoint Cox models to predict 1-, 2-, and 3-year overall survival in HCC. A total of 3850 patients diagnosed between 2018 and 2022 were included for model development and internal testing, and 2268 patients diagnosed in 2016–2017 formed an independent temporal validation cohort. Age, alpha-fetoprotein (AFP) level, fibrosis status, AJCC stage, and surgical treatment were identified as independent prognostic factors and incorporated into a multi-timepoint nomogram. The model achieved C-indices of 0.762, 0.760, and 0.726 in the training, test, and temporal validation cohorts. Time-dependent AUCs at 1, 2, and 3 years were 0.814, 0.801, and 0.780 in the training cohort; 0.824, 0.809, and 0.807 in the test cohort; and 0.794, 0.793, and 0.779 in the temporal validation cohort, demonstrating consistently strong discrimination. Calibration and decision curve analyses confirmed accurate agreement and clinical benefit. These findings indicate that the proposed model provides a robust tool for individualized survival prediction and risk-stratified clinical decision-making in HCC.

**Supplementary Information:**

The online version contains supplementary material available at 10.1038/s41598-026-48480-9.

## Introduction

Liver cancer is a major global health burden, ranking as the sixth most diagnosed malignancy and the third leading cause of cancer-related mortality. In 2022, approximately 870,000 new cases were reported worldwide, and projections indicate that the incidence may nearly double by 2050, exceeding 1.5 million newly diagnosed cases annually^1^. Hepatocellular carcinoma (HCC), the predominant form of primary liver cancer, accounts for nearly 80% of cases and is strongly associated with chronic hepatitis B or C infection, alcohol misuse, and metabolic disorders^2^. Due to its asymptomatic onset and high recurrence rate, many patients present at an advanced stage, and survival outcomes vary considerably even among those receiving curative or multimodal treatment^3,4^. Reliable and interpretable prognostic tools are therefore essential to support individualized clinical decision-making.

Commonly used prognostic systems for HCC, including the American Joint Committee on Cancer (AJCC), Barcelona Clinic Liver Cancer (BCLC), and China Liver Cancer Staging (CNLC) systems, are largely based on anatomical tumor burden and hepatic function, and thus have limited ability to capture biological and clinical heterogeneity across patients^5–7^. Recent advances in statistical modeling, artificial intelligence, and machine-learning approaches have enabled the integration of multidimensional clinical information to improve survival prediction^8,9^. However, many existing models suffer from important limitations: they are often derived from single-center cohorts without independent temporal or external validation, provide survival estimates at only a single timepoint, lack transparent reporting of sample size justification or events per variable, and may incorporate complex, non-interpretable modeling structures that hinder clinical applicability^10^. These limitations highlight the need for rigorously developed and validated Cox-based prognostic models capable of generating multi-timepoint survival predictions with high interpretability.

In this study, we used the population-based SEER registry to develop and temporally validate Cox proportional hazards models for predicting 1-, 2-, and 3-year overall survival in patients with HCC. A temporal split design was applied within the same database, using cases diagnosed from 2018 to 2022 for model development and internal testing, and cases from 2016 to 2017 as an independent temporal validation cohort to assess robustness across different time periods. Demographic, tumor-related, and treatment variables were evaluated using univariable and multivariable Cox regression, and model performance was assessed using the C-index, time-dependent AUC, calibration plots, and decision curve analysis. Risk stratification based on tertiles of log(HR) was further applied to enhance interpretability and facilitate potential clinical use.

## Methods

### Data source

This retrospective cohort study was conducted using data from the SEER program. Data were extracted with SEER*Stat software (version 9.0.41.4) from the dataset “Incidence – SEER Research Data, 17 Registries, Nov 2024 Sub (2000–2022) – Linked To County Attributes – Time Dependent (1990–2023) Income/Rurality, 1969–2023 Counties.” All data used in this study were based on the November 2024 submission and released in April 2025. The inclusion criteria were: (1) histology codes 8170/3–8175/3 according to ICD-O-3 (all subtypes of HCC); and (2) first malignant primary tumor. Restricting the cohort to first primary HCC cases was intended to minimize confounding from pre-existing malignancies at baseline. Patients with distant metastasis (M1) were not excluded, and metastatic status was incorporated as a prognostic variable in the model. Patients who developed subsequent primary malignancies after HCC diagnosis were not excluded, as these events occur after baseline and are inherently captured in overall survival. Exclusion criteria were: (1) cases identified solely through autopsy or death certificate; and (2) incomplete or non-calculable survival time.

For model development, patients diagnosed with HCC between 2018 and 2022 meeting the above criteria were first identified and randomly divided into a training cohort and a test cohort at a 7:3 ratio. To evaluate the temporal stability and generalizability of the model, an additional temporal validation cohort was constructed by extracting HCC cases diagnosed in 2016–2017 from the same SEER version using identical variable definitions, inclusion and exclusion criteria, and coding procedures. Apart from the diagnosis period, all other data elements and preprocessing steps were consistent across cohorts. This resulted in three analytical populations: (1) a training cohort (2018–2022, 70%) for model construction; (2) a test cohort (2018–2022, 30%) for internal performance assessment; and (3) a temporal validation cohort (2016–2017) for evaluating robustness across different time periods.

Access to the SEER database was granted in accordance with SEER program policies. All data used in this study were publicly available and de-identified; therefore, informed consent was not required. The study was reviewed by the Ethics Committee of the First Affiliated Hospital of Chongqing Medical University and was determined to be exempt from ethical review, with an official exemption certificate issued. All methods were performed in accordance with the relevant guidelines and regulations.

## Sample size estimation

To ensure adequate statistical power and model stability, sample size estimation was performed using the events-per-variable (EPV) approach. This study aimed to develop prognostic models for 1-, 2-, and 3-year overall survival (OS) in patients with HCC based on SEER data, with OS as the primary outcome. A total of 13 candidate predictors were planned for inclusion. Using the commonly recommended threshold of 10 events per variable (10 EPV) and assuming an anticipated event (death) rate of 0.45, the required sample size for the training cohort (70% of the total sample) was calculated to be approximately 289 patients.

To account for potential data loss due to incomplete records or missing follow-up information, an additional 10% buffer was applied. Therefore, a minimum total sample size of 459 patients was deemed necessary to meet the requirements for model development and validation.

## Variables

The following variables were extracted and analyzed in this study: (1) Demographic characteristics: sex, age, race, marital status, vital status, and survival time; (2) Tumor-related characteristics: T, N, and M stage and overall stage based on the AJCC 8th edition, alpha-fetoprotein (AFP) level, and fibrosis status; (3) Treatment-related information: surgery, radiotherapy, and chemotherapy. Age was categorized according to the optimal cutoff values determined in the training cohort. AFP levels were classified as negative (within the normal range) or positive (above the normal range). Fibrosis status was classified using the Ishak scoring system into non-cirrhosis (0–4) and cirrhosis (5–6). The selection of these variables was based on their biological plausibility and previously reported relevance to prognostic assessment in HCC^3,11,12^.

## Data processing

Data preprocessing and survival modeling were performed in Python (version 3.9 or higher), whereas baseline characteristic comparisons were conducted in R (version 4.2.1). The primary endpoint was overall survival (OS), defined as the interval from diagnosis to death from any cause; patients alive at last follow-up were censored. Continuous variables were standardized prior to modeling. Optimal age cutoffs were determined using X-tile software. Categorical variables were summarized as frequencies and percentages. Group comparisons were performed using the χ² test or Fisher’s exact test, as appropriate. To evaluate multicollinearity among categorical predictors, Cramér’s V coefficients were calculated, and variables with values > 0.7 were considered highly correlated and excluded from further modeling^13^. Remaining predictors were entered into multivariable Cox proportional hazards models to assess independent prognostic effects. Model development and evaluation—including variable selection, C-index estimation, time-dependent ROC analysis using the IPCW cumulative/dynamic method, calibration analysis, decision curve analysis, bootstrap resampling, risk score calculation, and Kaplan–Meier survival analysis with log-rank testing—were implemented in Python using standard scientific computing libraries. Cases with missing or non-informative values (e.g., “Unknown,” TX, NX) in key variables were excluded during cohort construction. A complete case analysis was performed without imputation. All tests were two-sided with a significance level of α = 0.01; *P* < 0.01 was considered statistically significant.

## Model development and evaluation

Independent prognostic factors were identified using multivariable Cox proportional hazards models and subsequently incorporated into a nomogram. Model discrimination was assessed using the concordance index (C-index) with corresponding 95% confidence intervals. Time-dependent areas under the ROC curve (AUCs) at 12, 24, and 36 months were calculated using the inverse probability of censoring weighting (IPCW) method, which applies weights derived from the estimated censoring distribution (e.g., Kaplan–Meier estimates) to reduce bias in sensitivity, specificity, and AUC calculations under right-censoring. Calibration performance at 1, 2, and 3 years was evaluated using calibration curves generated through bootstrap resampling (B = 1000), plotting predicted survival probabilities against observed outcomes to examine model fit across timepoints. Clinical utility was assessed using decision curve analysis (DCA), which quantified net benefit across a range of threshold probabilities. Confidence intervals for key performance metrics, including the C-index and time-dependent AUCs, were obtained through bootstrap resampling (B = 1000). All analyses followed a unified data preprocessing and coding strategy. The training, test, and temporal validation cohorts were strictly separated throughout the workflow to prevent information leakage.

### Risk stratification and survival analysis

Risk stratification was performed by calculating linear predictor values from the Cox proportional hazards model. Tertile-based cutoff points were determined through a grid-search procedure, evaluating candidate cutoff points within defined ranges and selecting the pair that maximized the log-rank statistic in the training cohort. This data-driven approach ensured optimal discrimination between risk groups while minimizing overfitting.

In addition to tertiles, we considered alternative methods for defining risk groups, including X-tile and maximally selected rank statistics. However, these methods tend to identify a single optimal cut point, which increases the risk of overfitting, particularly in retrospective datasets. In contrast, tertile-based stratification provides balanced group sizes, reduces overfitting, and enhances clinical interpretability. For these reasons, tertile-based stratification was chosen.

The cutoff values for low-, intermediate-, and high-risk groups were applied unchanged to both the test and temporal validation cohorts to ensure consistency and transferability. Kaplan–Meier survival curves were generated for each risk group, and both overall and pairwise log-rank tests were performed to assess survival differences. This approach allowed for a comprehensive evaluation of the discriminatory ability and robustness of the risk stratification method across different patient cohorts.

## Results

### Baseline clinical characteristics

**Fig. 1:**
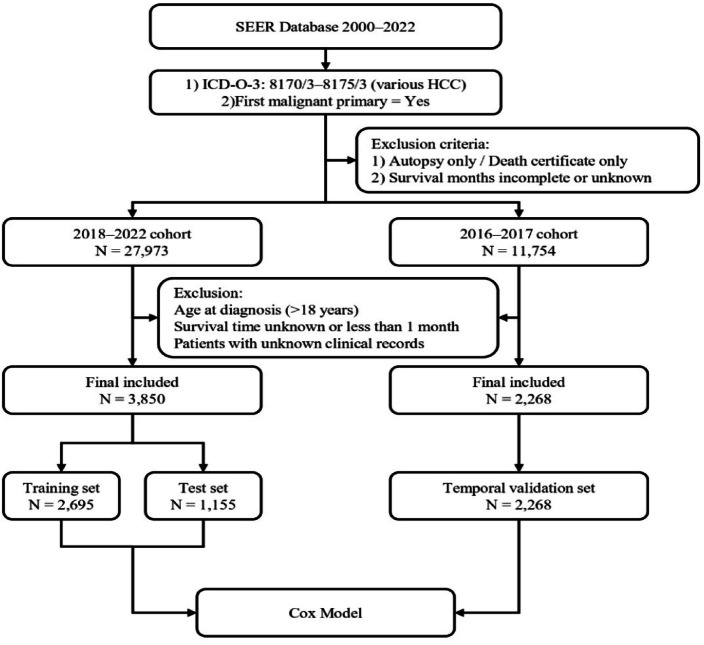
Flowchart of patient selection and cohort construction based on the SEER database (2000–2022).

Patients with HCC were first identified using ICD-O-3 histology codes 8170/3–8175/3, and only cases with HCC as the first malignant primary tumor were included. Cases identified solely through autopsy or death certificate, as well as those with incomplete or missing survival information, were excluded. Initial screening yielded 27,973 cases diagnosed between 2018 and 2022 and 11,754 cases diagnosed between 2016 and 2017. Additional exclusions were applied for age < 18 years at diagnosis, unknown survival time, survival duration < 1 month, or missing key clinical variables. A total of 3850 eligible patients from the 2018–2022 cohort were ultimately included for model development and testing and were randomly divided into a training cohort (*n* = 2,695) and a test cohort (*n* = 1,155). An independent temporal validation cohort comprising 2,268 patients diagnosed in 2016–2017 was also constructed (Fig. 1). The final sample size substantially exceeded the minimum required sample size of 459 patients, ensuring adequate statistical power for model development and validation. X-tile analysis identified 74 years as the optimal cutoff for age, which was subsequently applied for grouping in all cohorts.

In the overall development cohort (*n* = 3,850), 88.16% of patients were ≤ 74 years of age and 75.38% were male, consistent with the well-established higher incidence of HCC in men than in women in population-based studies^1,14^. Cirrhosis (Ishak score 5–6) was present in 80.08% of patients. According to the AJCC 8th edition staging system, 49.40% of cases were stage I, 22.62% stage II, 16.31% stage III, and 11.66% stage IV. Surgery, radiotherapy, and chemotherapy were administered in 40.52%, 26.52%, and 34.73% of patients, respectively.

For model development, patients diagnosed between 2018 and 2022 were randomly divided into a training cohort (*n* = 2,695) and a test cohort (*n* = 1,155) in a 7:3 ratio. As shown in Supplementary Table  S1 , no statistically significant differences were observed between the training and test cohorts across all baseline variables, confirming good internal comparability.

An independent temporal validation cohort comprising patients diagnosed between 2016 and 2017 (*n* = 2,268) was additionally constructed. Baseline characteristics of the three cohorts are presented in Table 1. Significant differences among the three cohorts were observed in age distribution, marital status, T stage, M stage, overall AJCC stage, radiotherapy, and chemotherapy (*P* < 0.01), whereas sex, race, AFP level, fibrosis status, N stage, and surgery were comparable among the cohorts.

**Table 1:** Baseline characteristics of patients in the training, test, and temporal validation cohorts.

Variables	Training (*n* = 2695)	Test (*n* = 1155)	Validation (*n* = 2268)	*P*
Age, n(%)				< 0.001
≤ 74	2384 (88.460)	1010 (87.446)	2051 (90.432)	
> 74	311 (11.540)	145 (12.554)	217 (9.568)	
Sex, n(%)				0.312
Female	673 (24.972)	275 (23.810)	523 (23.060)	
Male	2022 (75.028)	880 (76.190)	1745 (76.940)	
Race, n(%)				0.842
White	1871 (69.425)	817 (70.736)	1581 (69.709)	
Black	306 (11.354)	135 (11.688)	269 (11.861)	
Others	518 (19.221)	203 (17.576)	418 (18.430)	
Marital status, n(%)				0.004
Married	1499 (55.622)	648 (56.104)	1171 (51.631)	
Others	1196 (44.378)	507 (43.896)	1097 (48.369)	
AFP level, n(%)				0.832
Negative	1027 (38.108)	438 (37.922)	844 (37.213)	
Positive	1668 (61.892)	717 (62.078)	1424 (62.787)	
Fibrosis status, n(%)				0.215
Non-cirrhosis	546 (20.260)	221 (19.134)	478 (21.076)	
Cirrhosis	2149 (79.740)	934 (80.866)	1790 (78.924)	
T, n(%)				< 0.001
T1	1413 (52.430)	593 (51.342)	961 (42.372)	
T2	663 (24.601)	287 (24.848)	724 (31.922)	
T3	328 (12.171)	124 (10.736)	537 (23.677)	
T4	291 (10.798)	151 (13.074)	46 (2.028)	
N, n(%)				0.031
N0	2529 (93.840)	1073 (92.900)	2152 (94.885)	
N1	166 (6.160)	82 (7.100)	116 (5.115)	
M, n(%)				0.007
M0	2489 (92.356)	1049 (90.823)	2128 (93.827)	
M1	206 (7.644)	106 (9.177)	140 (6.173)	
Stage, n(%)				< 0.001
I	1342 (49.796)	560 (48.485)	924 (40.741)	
II	610 (22.635)	261 (22.597)	679 (29.938)	
III	442 (16.401)	186 (16.104)	451 (19.885)	
IV	301 (11.169)	148 (12.814)	214 (9.436)	
Surgery, n(%)				0.018
No	1596 (59.221)	694 (60.087)	1423 (62.743)	
Yes	1099 (40.779)	461 (39.913)	845 (37.257)	
Radiation, n(%)				< 0.001
No	1966 (72.950)	863 (74.719)	1951 (86.023)	
Yes	729 (27.050)	292 (25.281)	317 (13.977)	
Chemotherapy, n(%)				< 0.001
No	1752 (65.009)	761 (65.887)	1124 (49.559)	
Yes	943 (34.991)	394 (34.113)	1144 (50.441)	

### Univariable and multivariable analyses

**Fig. 2:**
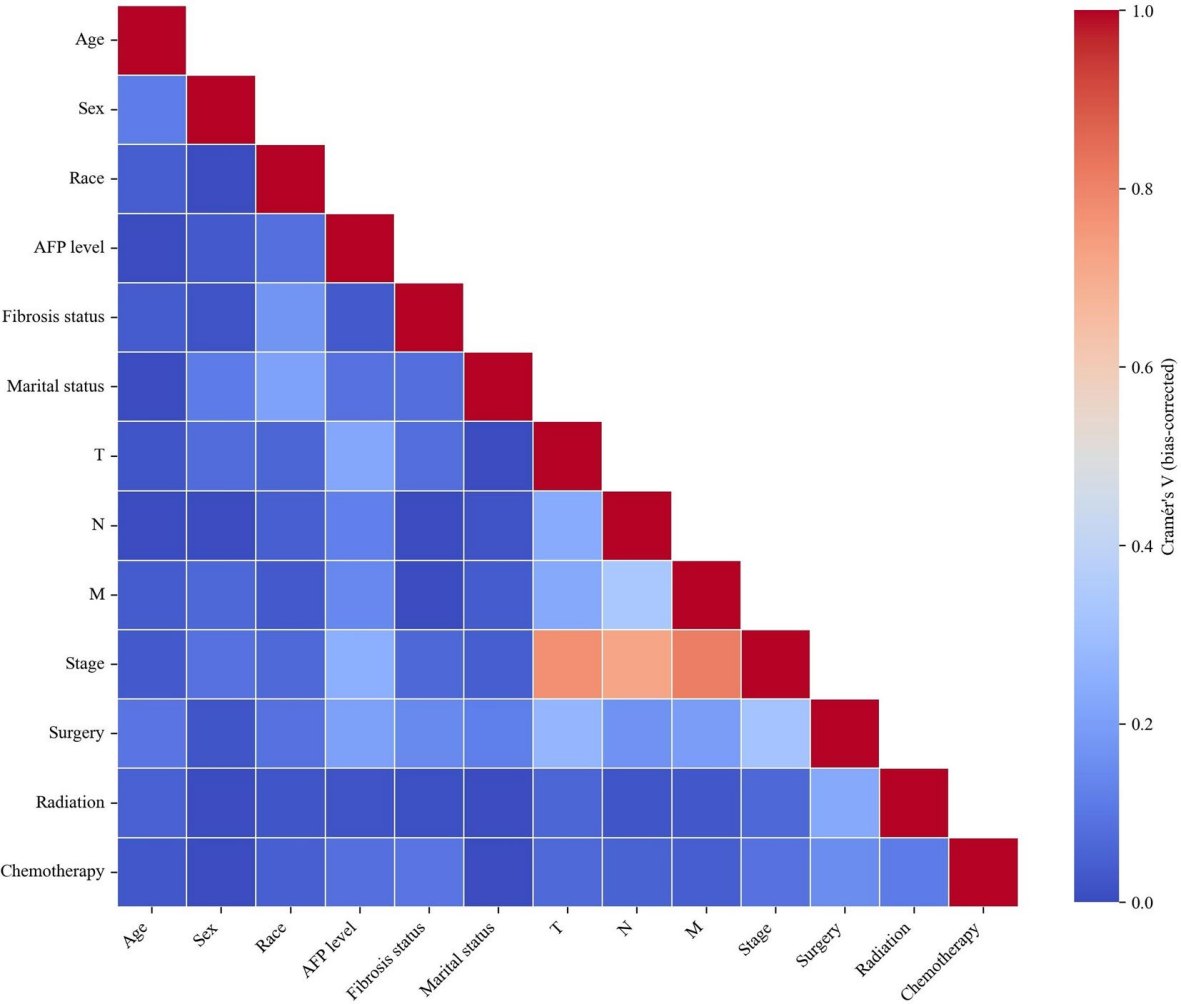
Heatmap of pairwise associations among variables using bias-corrected Cramér’s V.

Before regression modeling, we first assessed correlations among variables within the training cohort. Pairwise associations were quantified using bias-corrected Cramér’s V and visualized in a heatmap (Fig. 2). The results indicated strong correlations between overall stage and the T, N, and M stage (Cramér’s V > 0.7), suggesting substantial multicollinearity. Because AJCC stage is inherently defined by TNM components, including all these variables simultaneously would introduce redundancy and potentially destabilize parameter estimation. In accordance with the predefined threshold-based strategy, overall stage was retained while T, N, and M were excluded from further analyses.

**Fig. 3:**
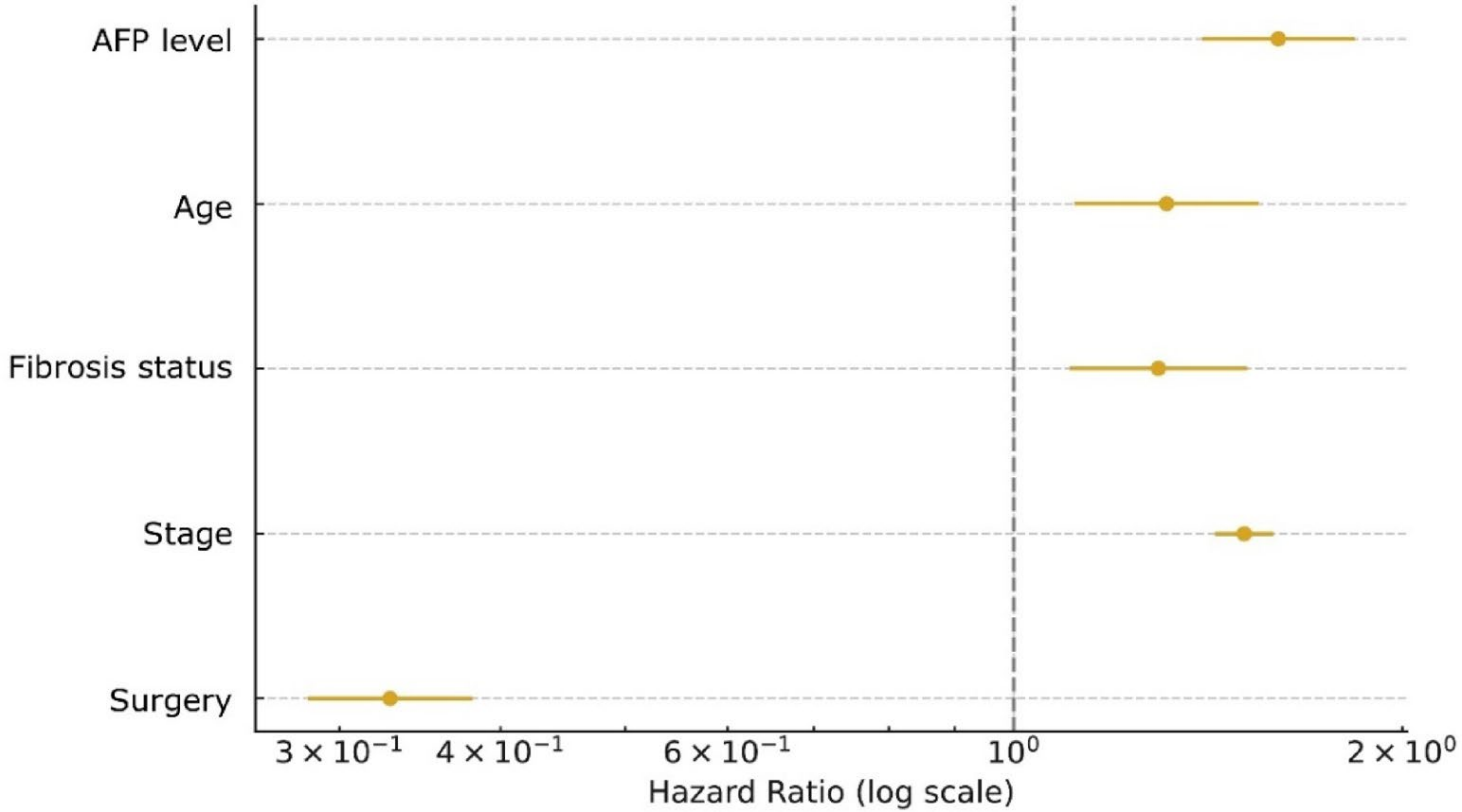
Forest plot of the final multivariable Cox proportional hazards model.

Subsequently, the remaining variables were entered into univariable and multivariable Cox regression analyses. Univariable results showed that age, marital status, race, AFP level, fibrosis status, surgical treatment, and AJCC stage were significantly associated with overall survival (all *P* < 0.01), whereas sex, chemotherapy, and radiotherapy did not reach statistical significance (all *P* > 0.01). Variables meeting the univariable threshold (*P* < 0.01) were included in the multivariable model, and the final set of predictors was determined based on the criterion of *P* < 0.01 in the multivariable analysis. The multivariable Cox model identified age (HR = 1.31, 95% CI 1.11–1.54, *P* < 0.01), AJCC stage (HR = 1.51, 95% CI 1.43–1.59, *P* < 0.01), AFP level (HR = 1.61, 95% CI 1.41–1.85, *P* < 0.01), and fibrosis status (HR = 1.31, 95% CI 1.12–1.53, *P* < 0.01) as independent risk factors for mortality. Surgical treatment was identified as an independent protective factor (HR = 0.33, 95% CI 0.28–0.38, *P* < 0.01). Marital status and race were not retained in the multivariable model (*P* > 0.01). The effect sizes of the final covariates were summarized in a forest plot (Fig. 3).

### Nomogram construction

**Fig. 4:**
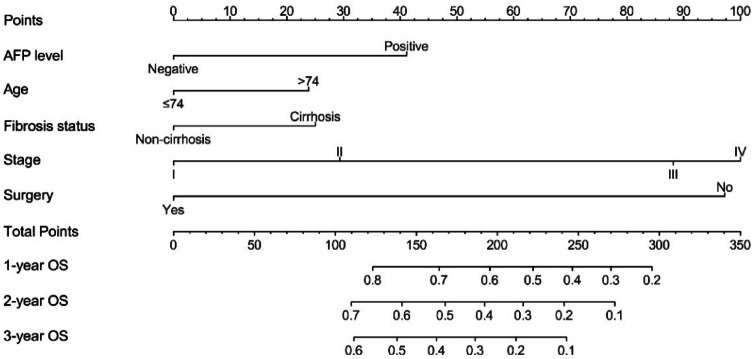
Nomogram for predicting 1-, 2-, and 3-year overall survival in patients with HCC.

Based on the independent prognostic factors identified in the multivariable Cox regression analysis—age, AJCC stage, AFP level, fibrosis status, and surgical treatment—a nomogram was developed to predict 1-, 2-, and 3-year overall survival (OS) (Fig. 4). The regression coefficients of each covariate were proportionally converted into point values, and individual total scores were calculated by summing the weighted points. These total scores were then mapped to the corresponding predicted probabilities of 1-, 2-, and 3-year OS.

### Model performance evaluation

**Fig. 5:**
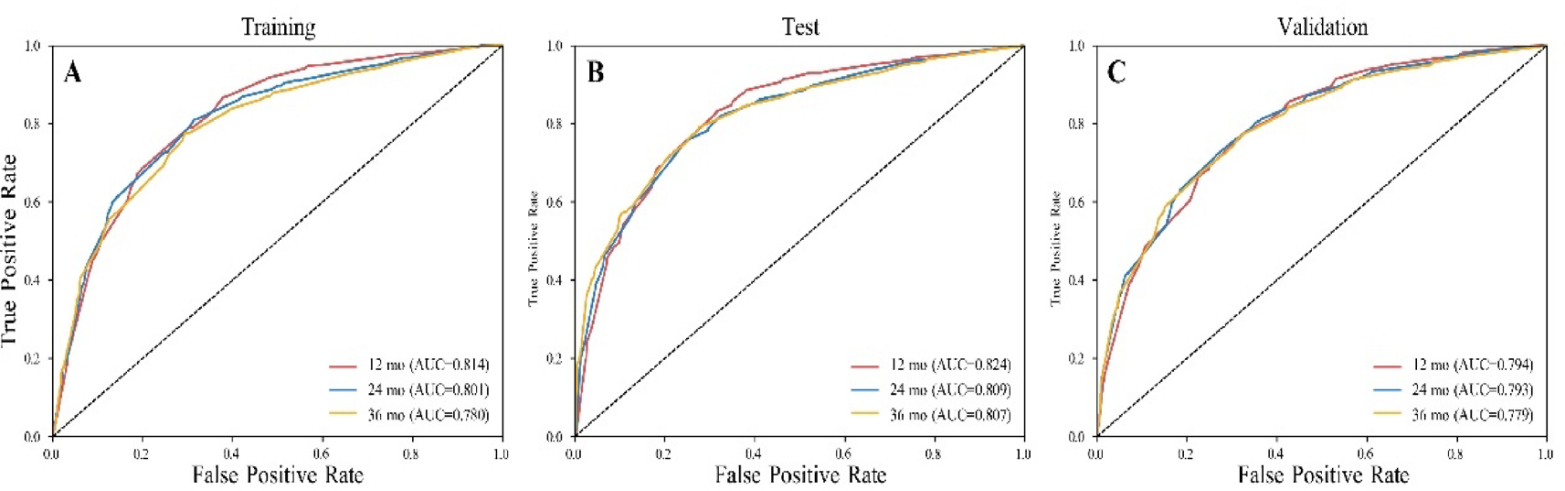
Time-dependent ROC curves of the prognostic model at 12, 24, and 36 months in the training (**A**), test (**B**), and temporal validation (**C**) cohorts.

**Fig. 6:**
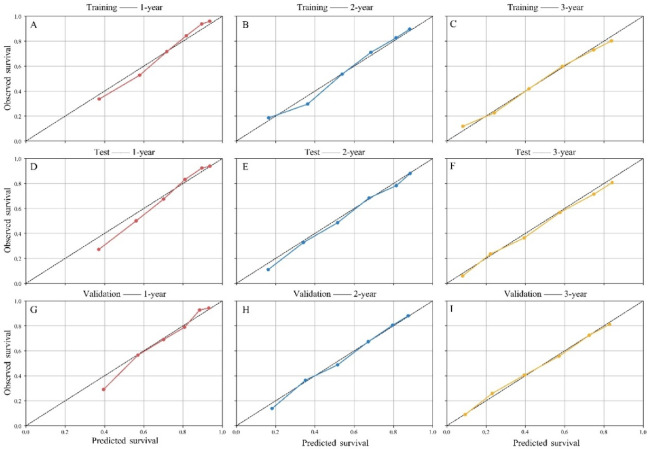
Calibration curves for predicting 1-, 2-, and 3-year overall survival in the training (**A**–**C**), test (**D**–**F**), and temporal validation (**G**–**I**) cohorts.

**Fig. 7:**
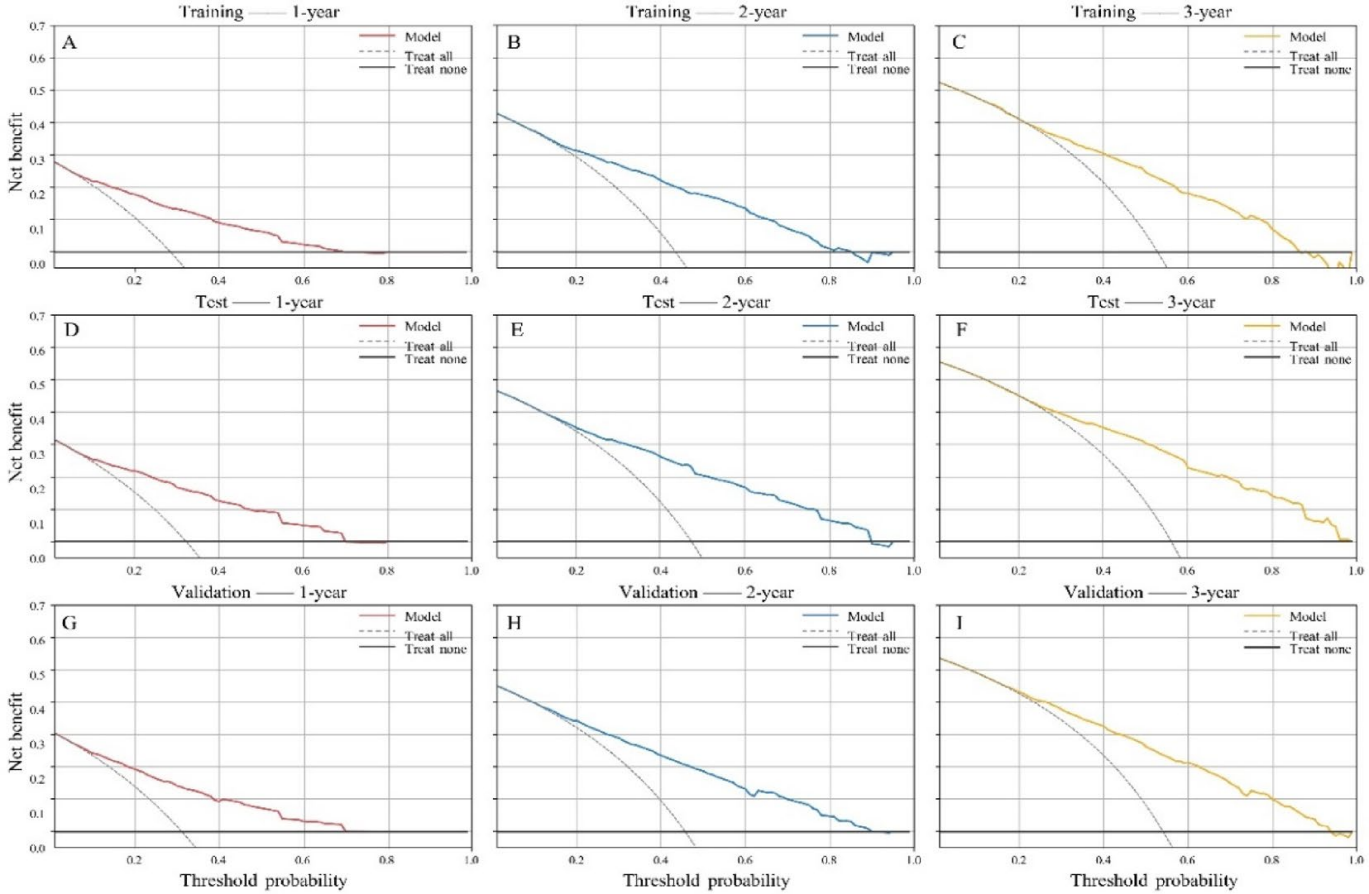
Decision curve analysis (DCA) for predicting 1-, 2-, and 3-year overall survival in the training (**A**–**C**), test (**D**–**F**), and temporal validation (**G**–**I**) cohorts.

The nomogram demonstrated good discriminatory performance. The C-index was 0.762 (95% CI: 0.750–0.775) in the training cohort, 0.760 (95% CI: 0.742–0.780) in the test cohort, and 0.726 (95% CI: 0.714–0.738) in the temporal validation cohort. Time-dependent ROC analyses further confirmed the high accuracy and discriminative ability of the model. The AUCs for predicting 1-, 2-, and 3-year overall survival were 0.814, 0.801, and 0.780 in the training cohort; 0.824, 0.809, and 0.807 in the test cohort; and 0.794, 0.793, and 0.779 in the temporal validation cohort (Fig. 5). These findings indicate that the model maintains strong discrimination across multiple timepoints and demonstrates consistent performance in temporal validation, supporting its generalizability across time. Additionally, calibration curves showed good agreement between predicted and observed survival probabilities (Fig. 6). Decision curve analysis was used to evaluate the clinical utility of the model by quantifying net benefit across a range of threshold probabilities, which represent the probability at which a clinician would choose to intervene (e.g., intensified surveillance, adjuvant therapy, or closer follow-up). The model demonstrated higher net benefit than both the “treat-all” and “treat-none” strategies across clinically relevant threshold ranges. For 1-year survival prediction, the model showed superior net benefit approximately between threshold probabilities of 0.05 and 0.60. This suggests that for patients whose predicted risk of death falls within this range, using the model to guide clinical decisions would yield more appropriate intervention allocation than uniformly treating all patients or none. At the 2- and 3-year timepoints, the range of net benefit extended to approximately 0.80–0.85, indicating that the model remains informative even at relatively high-risk thresholds. Similar decision curve patterns were observed across the training, test, and temporal validation cohorts, indicating consistent clinical utility despite differences in baseline characteristics. (Fig. 7).

### Cox-based risk stratification

**Fig. 8:**
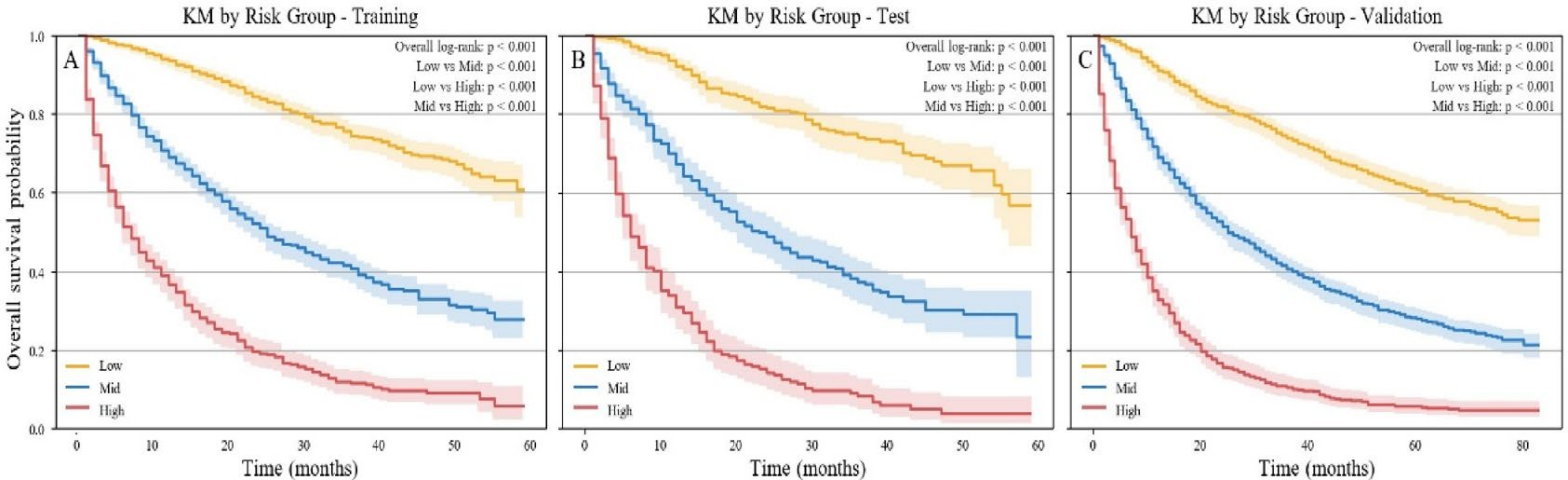
Kaplan–Meier survival curves for low-, mid-, and high-risk groups in the training (**A**), test (**B**), and temporal validation (**C**) cohorts.

The individual risk score was defined as the log partial hazard (log-HR) derived from the final Cox proportional hazards model in the training cohort. Two cutoff points (− 0.180 and 0.853) were identified within the training cohort by maximizing the log-rank statistic over a predefined grid. Based on these thresholds, patients were categorized into low-, intermediate-, and high-risk groups. As shown in Fig. 8A, the Kaplan–Meier curves demonstrated statistically significant differences among the three groups (*P* < 0.001). Similar stepwise separation of survival curves was observed in both the test cohort and the temporal validation cohort (Fig. 8B and C ), indicating the strong risk-stratification ability of the Cox model.

## Discussion

HCC remains a highly heterogeneous malignancy with substantial variation in etiology, tumor biology, and clinical course. Its global burden has continued to rise in recent years^14^. Due to its insidious onset, rapid progression, and high recurrence rate, many patients are diagnosed at an advanced stage, resulting in generally poor survival outcomes. Conventional staging systems, including AJCC, BCLC, and CNLC, provide structured frameworks for risk stratification but capture only limited dimensions of prognostic variability. In this context, prognostic models that integrate multidimensional clinical information and generate dynamic survival estimates across multiple timepoints may better support individualized risk assessment and clinical decision-making.

HCC management is inherently stage-dependent and multimodal. While curative treatments are preferred for early-stage disease, locoregional and systemic therapies are widely applied in intermediate and advanced settings. However, population-based registries such as SEER do not comprehensively capture detailed procedural or regimen-specific information. Therefore, the present study incorporated treatment variables consistently available in SEER—surgery, radiotherapy, and chemotherapy—as proxies for major therapeutic pathways. Inclusion of these variables allows the model to partially account for survival heterogeneity associated with treatment strategies while maintaining applicability within a large real-world dataset.

In this study, we developed and validated a multi-timepoint (1-, 2-, and 3-year) overall survival prediction model for HCC using the large, population-based SEER registry. The model demonstrated consistent discriminatory performance with C-indices of 0.762 in the training cohort, 0.760 in the test cohort, and 0.726 in the temporal validation cohort. Time-dependent ROC curves, calibration plots, and decision curve analysis further supported its accuracy, reliability, and clinical utility. The model’s risk stratification system, based on tertile-based cutoffs, provides a simple, interpretable tool for clinical management of HCC. This study presents a robust framework for individualized prognosis and risk assessment, with potential for future external validation and clinical translation.

Although the training and test cohorts had comparable baseline characteristics as a result of random splitting, the temporal validation cohort showed differences in tumor stage distribution and treatment patterns. In particular, the validation cohort included a higher proportion of T2–T3 and stage II–III cases, as well as changes in the use of radiotherapy and chemotherapy. These variations may be related to temporal changes in clinical practice and disease presentation. Despite these differences, the model maintained similar discrimination and calibration performance in the temporal validation cohort. This finding suggests that the model retains predictive stability across populations with different baseline characteristics, supporting its potential generalizability.

In previous research, substantial efforts have been made to develop survival prediction models for HCC. For example, a recent systematic review reported that several prognostic models have been validated for postoperative survival prediction; however, most demonstrated suboptimal discriminatory performance in external validation, with C-indices typically below 0.70, and the overall methodological quality of the studies varied considerably^15^. In addition, for patients with HCC receiving systemic therapies such as targeted agents or immunotherapy, existing studies have similarly reported that most available prognostic models rely solely on internal validation, are based on relatively small sample sizes, and incorporate limited clinical variables, with few models undergoing temporal or multicenter external validation^16^. In recent years, machine-learning and deep-learning approaches have been introduced for survival prediction in HCC. However, many of these models are derived from single-center cohorts, involve limited sample sizes, lack the ability to provide multi-timepoint predictions (e.g., 1-, 2-, and 3-year survival), or offer limited interpretability, which restricts their applicability in clinical practice^17,18^. Compared with previous studies, this work offers methodological innovations and practical value. First, the model was developed using the large, population-based SEER registry, which includes data from multiple regions and medical centers across the United States. The large sample size and substantial population heterogeneity provide a robust epidemiological foundation for model development and help overcome limitations of prior studies that were often single-center, small-scale, or insufficiently validated. Second, the present study incorporated a multi-timepoint prediction framework, estimating 1-, 2-, and 3-year overall survival, which allows the model to capture both the natural history of the disease and dynamic changes associated with antitumor treatment. This enhances the model’s temporal relevance and practical value for clinical decision-making. In addition, a rigorous temporal validation design was implemented by applying a non-random time-split based on diagnosis year (2018–2022 for model development and testing, and 2016–2017 for temporal validation). This approach enabled assessment of model performance and generalizability across different time periods, demonstrating temporal robustness and addressing a common limitation of existing models that rely solely on internal validation within a single time window. Furthermore, risk stratification into low-, intermediate-, and high-risk groups was achieved using tertile-based cutoffs of the log hazard ratio, providing not only quantitative survival predictions but also an interpretable clinical risk classification system. This facilitates identification of higher-risk patients and may assist clinicians in tailoring surveillance and management strategies. Taken together, the model developed in this study demonstrates strong predictive accuracy, temporal adaptability, and clinical applicability. It has the potential to serve as a valuable supplementary tool for risk stratification and decision support in the comprehensive management of patients with HCC.

The present study was conducted within a prognostic prediction framework rather than a causal inference framework. Although demographic and clinical variables such as race, sex, tumor stage, and treatment may influence survival through complex intermediary pathways, the model was designed to estimate overall risk rather than to decompose direct and indirect effects. Formal mediation analysis requires clearly defined temporal relationships and comprehensive adjustment for potential confounders, which may not be fully supported in registry-based retrospective datasets such as SEER. Future studies applying causal modeling approaches may further elucidate potential mechanistic pathways underlying survival disparities.

Despite its methodological strengths, this study has several limitations. First, although SEER provides a large population-based cohort, its retrospective nature introduces potential selection bias and incomplete information. The database lacks key clinical variables relevant to HCC prognosis, including liver function parameters, Child–Pugh classification, tumor biomarkers, molecular profiling, post-treatment dynamics, and detailed information on local and systemic therapies^19–22^. Second, SEER does not capture detailed treatment regimens, treatment response, recurrence, or progression data, and is subject to inherent delays in data updates. Variations in follow-up practices and data completeness may further introduce heterogeneity, potentially affecting generalizability^18,23^. Third, although temporal validation was performed, the validation cohort was derived from the same registry using a time-split approach rather than an independent external dataset. Moreover, the reverse temporal validation design—using earlier cases to validate a model developed from more recent data—does not fully replicate prospective deployment. Substantial regional and racial differences in etiology and healthcare resources may also limit broader applicability. Prospective, multicenter external validation remains necessary^24,25^. Furthermore, although nomograms offer favorable interpretability and visual clarity, they inherently rely on predefined linear assumptions and a fixed set of variables. When the distribution of clinical characteristics or treatment patterns changes substantially in real-world practice, predictive performance may deteriorate, necessitating periodic recalibration or model updating. Consistent with this concern, previous studies have reported that traditional nomogram models often require parameter re-optimization when applied to heterogeneous populations to prevent overfitting or loss of accuracy^26,27^. Finally, overall survival was used as the primary endpoint, while other clinically relevant outcomes, such as cancer-specific survival and recurrence-free survival, were not evaluated. Future studies incorporating prospective real-world data and multiple outcome dimensions are warranted to further refine and validate the model.

Future research should focus on validating this model in larger and more diverse cohorts. Integration of molecular, imaging, and metabolic data may further improve predictive performance and biological relevance. Implementation through digital tools or electronic health record systems could facilitate clinical application.

In summary, we developed and temporally validated a multi-timepoint survival prediction model for HCC using a large population-based dataset. The model demonstrated stable discrimination, calibration, and decision-curve performance across cohorts. While further external validation is required, this work provides a structured framework for risk stratification in HCC.

## Supplementary Information

Below is the link to the electronic supplementary material.

Supplementary Material 1

## Data Availability

The data supporting this study are publicly accessible from the SEER Program. Access to public-use SEER datasets requires registration and acceptance of the SEER Research Data Agreement through the SEER*Stat data portal (https://seer.cancer.gov/data/). The dataset used was Incidence – SEER Research Data, 17 Registries, Nov 2024 Submission (2000–2022), released April 2025. Documentation for this submission is available at: https://seer.cancer.gov/data-software/documentation/seerstat/nov2024/.
