# Supplementary material for: Development and temporal validation of 1-, 2-, and 3-year survival prediction models for hepatocellular carcinoma using the SEER database
Source: Sci Rep. 2026 Apr 11;16:16443. doi: 10.1038/s41598-026-48480-9 (PMC13216628; doi:10.1038/s41598-026-48480-9)
Supplement: Supplementary file 1 — Supplementary Material 1 [file 41598_2026_48480_MOESM1_ESM.pdf]

Supplementary Table S1. Main Characteristic Distribution of Data in the Training Cohorts and the Test Cohorts

**Variables**	**Total (n = 3850)**	**Training (n = 2695)**	**Test (n = 1155)**	**P**
Age, n(%)				0.372
≤74	3394 (88.156)	2384 (88.460)	1010 (87.446)	
>74	456 (11.844)	311 (11.540)	145 (12.554)	
Sex, n(%)				0.443
Female	948 (24.623)	673 (24.972)	275 (23.810)	
Male	2902 (75.377)	2022 (75.028)	880 (76.190)	
Race, n(%)				0.485
White	2688 (69.818)	1871 (69.425)	817 (70.736)	
Black	441 (11.455)	306 (11.354)	135 (11.688)	
Others	721 (18.727)	518 (19.221)	203 (17.576)	
Marital status, n(%)				0.782
Married	2147 (55.766)	1499 (55.622)	648 (56.104)	
Others	1703 (44.234)	1196 (44.378)	507 (43.896)	
AFP, n(%)				0.913
Negative	1465 (38.052)	1027 (38.108)	438 (37.922)	
Positive	2385 (61.948)	1668 (61.892)	717 (62.078)	
Fibrosis, n(%)				0.423
Non-cirrhosis	767 (19.922)	546 (20.260)	221 (19.134)	
Cirrhosis	3083 (80.078)	2149 (79.740)	934 (80.866)	
T, n(%)				0.153
T1	2006 (52.104)	1413 (52.430)	593 (51.342)	
T2	950 (24.675)	663 (24.601)	287 (24.848)	
T3	452 (11.740)	328 (12.171)	124 (10.736)	
T4	442 (11.481)	291 (10.798)	151 (13.074)	
N, n(%)				0.276
N0	3602 (93.558)	2529 (93.840)	1073 (92.900)	
N1	248 (6.442)	166 (6.160)	82 (7.100)	
M, n(%)				0.110
M0	3538 (91.896)	2489 (92.356)	1049 (90.823)	
M1	312 (8.104)	206 (7.644)	106 (9.177)	
Stage, n(%)				0.532
I	1902 (49.403)	1342 (49.796)	560 (48.485)	
II	871 (22.623)	610 (22.635)	261 (22.597)	
III	628 (16.312)	442 (16.401)	186 (16.104)	
IV	449 (11.662)	301 (11.169)	148 (12.814)	
Surgery, n(%)				0.616
No	2290 (59.481)	1596 (59.221)	694 (60.087)	
Yes	1560 (40.519)	1099 (40.779)	461 (39.913)	
Radiation, n(%)				0.255
No	2829 (73.481)	1966 (72.950)	863 (74.719)	
Yes	1021 (26.519)	729 (27.050)	292 (25.281)	
Chemotherapy, n(%)				0.600
No	2513 (65.273)	1752 (65.009)	761 (65.887)	
Yes	1337 (34.727)	943 (34.991)	394 (34.113)	
